# Allergenic Responses Elicited by Goat Milk Protein Hydrolysates in Cow Milk-Sensitized Mice

**DOI:** 10.3390/ijms27209230

**Published:** 2026-10-17

**Authors:** Wenxuan Zhao, Sihan Lu, Yihan Liu, Pengmei An, Yanjun Cong

**Affiliations:** College of Food and Health, Beijing Technology and Business University, Beijing 100048, China; 18634443697@163.com (W.Z.); 13102635808@163.com (S.L.); 18242397133@163.com (Y.L.); an18701249218@163.com (P.A.)

**Keywords:** goat milk protein, protease, hydrolysate, elicitation response, mouse model

## Abstract

This study established a BALB/c mouse model of milk allergy to investigate the allergic responses elicited by goat milk protein hydrolysates prepared using three proteases—alkaline protease, bromelain, and trypsin—in sensitized mice. Serum-specific IgG and total IgE levels were measured, and allergic symptom scores and histopathological changes were evaluated. The results showed significant differences in clinical symptom scores among the different treatment groups (*p* < 0.05), with a significant difference observed between the bromelain hydrolysate group and the GM group (*p* < 0.05). Serum-specific IgG and total IgE levels were also significantly lower in all three hydrolysate groups than in the whole goat milk protein group (*p* < 0.05), with the lowest specific IgG level observed in the trypsin hydrolysate group. The bromelain hydrolysate group exhibited the lowest clinical symptom score and serum histamine level. Histopathological analysis further confirmed that, compared with the whole goat milk protein group, the hydrolysate groups exhibited less intestinal mucosal damage and reduced splenic congestion. In summary, this study evaluated the elicitation response to goat milk protein hydrolysates in cow milk protein-sensitized mice. The three hydrolysates elicited weaker allergic responses than total goat milk protein. However, because the hydrolysates were administered only during the challenge phase, their intrinsic sensitizing capacity was not assessed. These findings provide a basis for further investigation into the potential use of hydrolysates in hypoallergenic goat milk-based formula foods, which will require additional studies.

## 1. Introduction

Milk and dairy products are important sources of dietary protein, and milk consumption accounts for approximately 95% of dairy product consumption [1]. However, milk proteins are also among the most common food allergens in infants and children. More than 20 distinct milk proteins have been reported to elicit allergic responses in susceptible individuals. The prevalence of cow’s milk allergy (CMA) in children younger than 3 years is reported to be approximately 8% [2]. CMA can cause allergic dermatitis, diarrhea, vomiting, dyspnea, and other symptoms. In severe cases, it can lead to anaphylactic shock and even life-threatening reactions. Milk allergy is primarily caused by an abnormal immune response to milk proteins, among which casein, β-lactoglobulin, and α-lactalbumin exhibit potential allergenicity [3]. β-Lactoglobulin accounts for approximately 50% of whey proteins, whereas casein accounts for approximately 80% of the total protein content in milk [4]. The entry of allergens into the body triggers an IgE-mediated immune response [5], resulting in the production of specific IgE antibodies [6]. These antibodies bind to mast cells and basophils and, upon subsequent exposure to allergens, induce the release of histamine and other pro-inflammatory mediators, leading to cutaneous, gastrointestinal, and systemic allergic symptoms. Dietary avoidance of milk and its derivatives remains a cornerstone of CMA management, although prolonged dietary restriction may compromise the adequate intake of high-quality protein and essential micronutrients [7]. Therefore, the development of low-allergenicity milk protein sources and related food ingredients is of significant nutritional and practical value.

Goat milk is nutritionally rich and contains a variety of bioactive compounds, including growth factors, oligosaccharides, and bioactive peptides [8]. It has been reported to exhibit antioxidant, hypocholesterolemic, and gut-modulatory properties [9]. In addition, the secondary structure of goat milk whey proteins closely resembles that of human milk whey proteins, which may contribute to their favorable digestibility and absorption [10]. Bioactive peptides generated through the hydrolysis of goat milk proteins exhibit multiple beneficial activities, including antioxidant, antimicrobial, and antihypertensive effects. Furthermore, owing to its distinctive protein profile and physicochemical characteristics, goat milk has been recognized as a promising alternative to bovine milk in infant formula and dairy products [11]. Compared with bovine milk proteins, goat milk proteins not only meet the nutritional requirements of infants and young children but may also exhibit lower allergenic potential. Moreover, goat milk β-lactoglobulin is more susceptible to enzymatic degradation in the small intestine than its bovine counterpart [12]. The consumption of goat milk and its derivatives has steadily increased in recent years [13]. Furthermore, studies using an in vitro infant digestion model have demonstrated that goat milk-based infant formula exhibits higher protein digestibility than bovine milk-based formula [14]. It has also been reported that some individuals with cow’s milk protein allergy can tolerate goat milk to some extent [15]. Consequently, goat milk and its protein fractions have attracted increasing attention as potential alternatives for the development of hypoallergenic dairy products. However, goat milk is not completely free of allergenic potential [16]. It contains multiple proteins with immunogenic potential, and certain goat milk proteins exhibit substantial amino acid sequence and structural similarity to bovine milk proteins. Studies have demonstrated that bovine and goat milk αs1-casein share up to 89% amino acid sequence identity [17]. Despite differences in some individual epitopes, the two proteins have similar overall epitope numbers and exhibit partial overlap in their antigenic epitope regions [18]. Consequently, potential cross-reactivity exists between bovine and goat milk proteins. Several case reports have indicated that some individuals with cow’s milk allergy remain at risk of adverse reactions following the consumption of goat milk [19]. Thus, the purported “low allergenic potential” of goat milk does not necessarily guarantee its safety for individuals with cow’s milk allergy. Therefore, developing goat milk products with reduced allergenicity for individuals with cow’s milk allergy is crucial for improving the safety of goat milk proteins.

Protein hydrolysis is an important approach for reducing the allergenicity of milk proteins. Proteolytic hydrolysis can disrupt the structure of intact proteins and degrade macromolecular proteins into peptides or amino acids with different molecular weights, thereby altering or destroying antigenic epitopes [20], reducing their ability to bind to specific IgE, and consequently decreasing their potential to induce allergic reactions. Numerous studies have demonstrated that enzymatic hydrolysis can effectively reduce the allergenicity of milk proteins, and this approach has been applied in the production of partially hydrolyzed and extensively hydrolyzed infant formulas [21]. At present, the reduced allergenicity of milk protein hydrolysates is mainly evaluated using alkaline or combined enzymatic hydrolysis [22]. A combination of molecular weight distribution analysis, IgE-binding assays, and cellular or animal models is commonly used to evaluate residual allergenicity. However, conventional proteolytic hydrolysis is inherently nonspecific, and it is difficult to ensure the effective elimination of key immune-recognition epitopes based solely on the degree of hydrolysis or molecular weight distribution. Furthermore, peptide fragments generated during hydrolysis may retain residual immunoreactivity. Therefore, the identification and selective elimination of key immunogenic epitopes in milk proteins represent important strategies for improving the safety of milk protein hydrolysates. Previous studies have identified three proteases—alkaline protease, bromelain, and trypsin—that can effectively reduce cross-reactivity, as demonstrated by in vitro serological assays. Bioinformatics analyses have further confirmed that major allergenic proteins in bovine and goat milk share high amino acid sequence similarity and common cross-reactive linear epitopes, providing a molecular basis for potential cross-sensitization between bovine and goat milk [23]. However, reduced antigenicity observed in vitro does not necessarily reflect the actual allergenic potential or immunogenicity of hydrolysates in vivo. Therefore, further evaluation of their in vivo safety and immunomodulatory properties using appropriate animal models is essential.

Animal models serve as invaluable tools for assessing the allergenicity of food proteins because they recapitulate the physiological processes underlying allergic responses, from immune activation during the sensitization phase to effector cell degranulation during the challenge phase [24]. BALB/c mice are well-established models for allergy research and are characterized by a Th2-skewed immune response and high susceptibility to oral sensitization; consequently, they have been extensively used in studies of cow’s milk protein allergy [25]. In this study, we used a cow milk protein-sensitized mouse model to compare the allergic responses elicited by intact goat milk protein and its enzymatic hydrolysates during the challenge phase. By monitoring allergy-related clinical signs, including changes in body temperature, we evaluated their ability to induce specific antibody production, the severity of clinical symptoms following challenge, and pathological changes in target organs, such as the intestine and spleen. These parameters were used to characterize the allergic responses elicited by the hydrolysates in sensitized mice and provide an experimental basis for further investigation into the potential use of goat milk protein hydrolysates in hypoallergenic formula foods.

## 2. Results

### 2.1. Changes in Body Weight of Mice in Each Group

**Figure 1:**
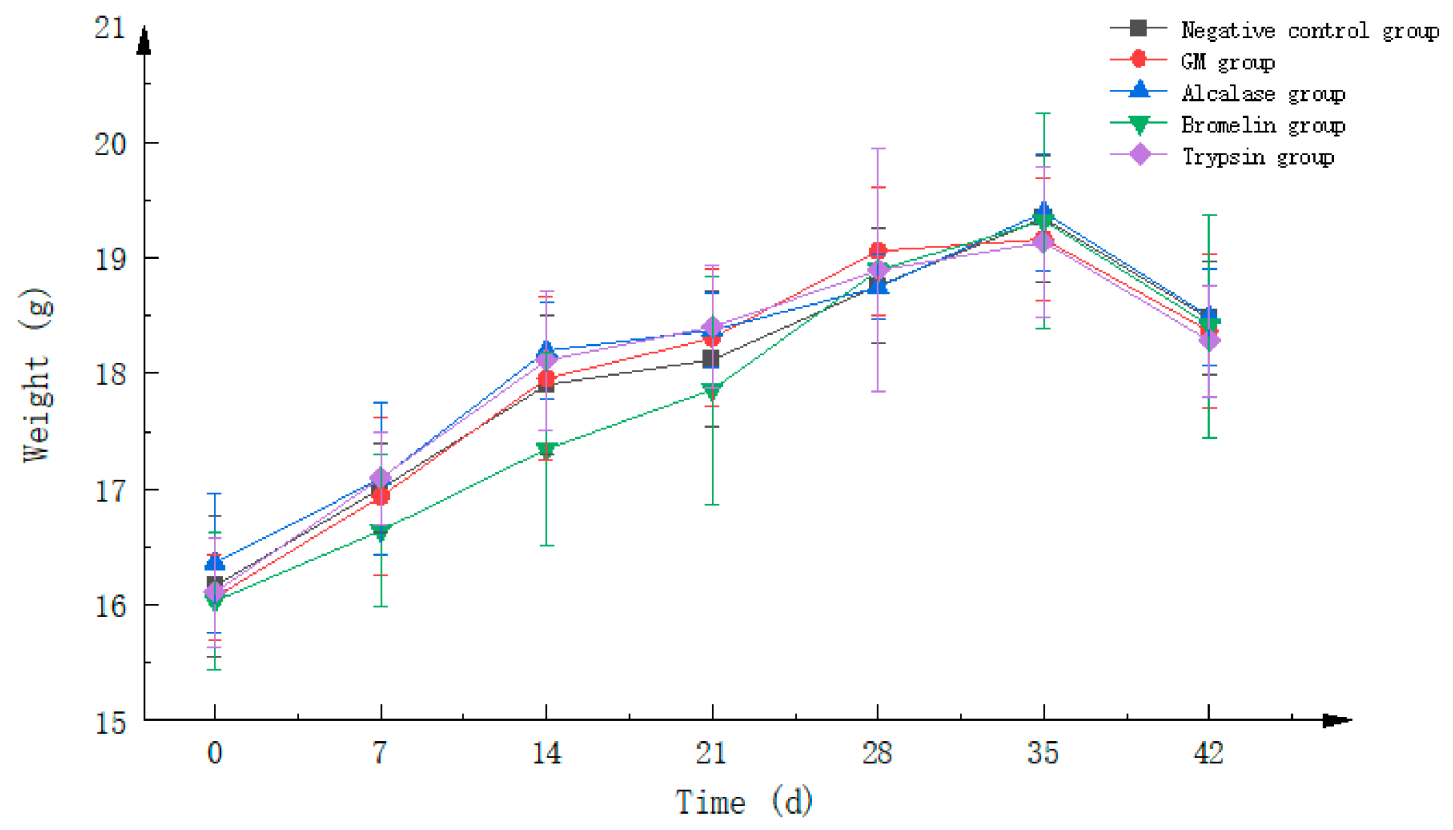
Changes in body weight of mice in each group during the experimental period. Sample size *n* = 10.

Figure 1 shows that mice in all groups exhibited continuous weight gain throughout the experimental period, indicating that the gavage treatments did not significantly affect their normal growth and development. At the beginning of the experiment (day 0), the mean body weight of the mice was 16.4 g, with no significant differences observed among the groups. From day 7 onward, distinct patterns of body weight changes emerged among the treatment groups. The negative control group (NC) exhibited the most consistent weight gain throughout the study, with body weight increasing steadily from 16.4 g on day 0 to 18.8 g on day 42, corresponding to a cumulative gain of 2.4 g. The body weight trajectories of mice in the goat milk total protein group (GM) and the three hydrolysate groups were generally comparable to those of the NC group, although slightly lower values were observed at some time points. By the end of the experiment (day 42), the body weights of mice in the GM group and all hydrolysate groups were comparable to those of the NC group (approximately 18.8 g). From day 14 to day 35, the body weights of mice in the treatment groups ranged from 17.2 to 19.6 g, which was slightly lower than the range observed in the NC group (18.0–19.6 g) during the same period; however, these differences were not statistically significant, and the mice maintained normal physiological growth trajectories. Overall, no abnormal phenomena, such as weight loss or growth retardation, were observed in any group throughout the experimental period.

### 2.2. Clinical Symptoms of Mice in Each Group

On day 42, all groups of mice were subjected to the challenge, and their clinical symptoms were recorded. The data were analyzed using the Kruskal–Wallis H non-parametric test. As shown in Table 1, significant differences in symptom scores were observed among the different treatment groups (H = 17.740, df = 4, *p* = 0.001387 < 0.05).

**Figure 2:**
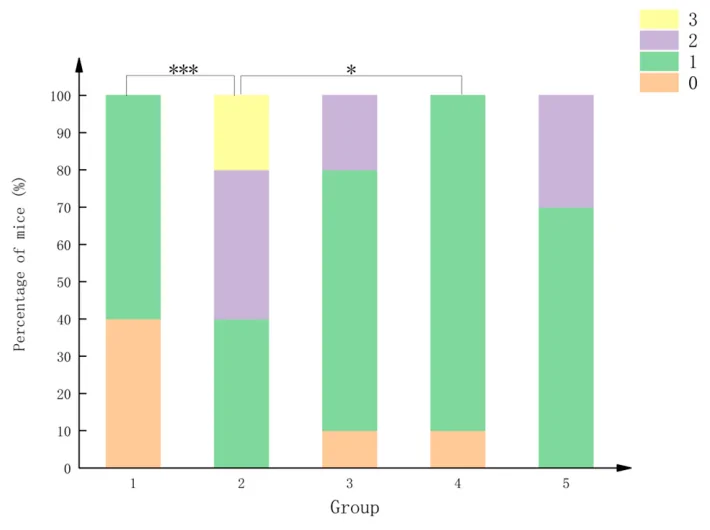
Percentage stacked bar chart of allergic symptom scores in mice from different treatment groups. Note: 1—Negative control group, 2—Goat milk total protein group (GM), 3—Alkaline protease hydrolysate group, 4—Bromelain hydrolysate group, 5—Trypsin hydrolysate group. Asterisks indicate the statistical significance of between-group comparisons. (* *p* < 0.05, *** *p* ≤ 0.001).

As shown in Figure 2, different colors represent different levels of symptom severity. Mice in the negative control (NC) and bromelain hydrolysate groups exhibited relatively mild allergic symptoms, with scores predominantly concentrated at 0 and 1. In contrast, mice in the goat milk total protein (GM) group exhibited the most severe allergic symptoms, with a higher proportion of mice receiving scores of 2 or 3, supporting the successful establishment of the allergy model. To further determine the statistical differences between groups, post hoc pairwise comparisons were performed using Dunn’s test with Bonferroni correction. The results revealed a significant difference between the bromelain hydrolysate group and the GM group (*p* = 0.043 < 0.05).

### 2.3. Results of Apecific Antibody IgG Measurement

**Figure 3:**
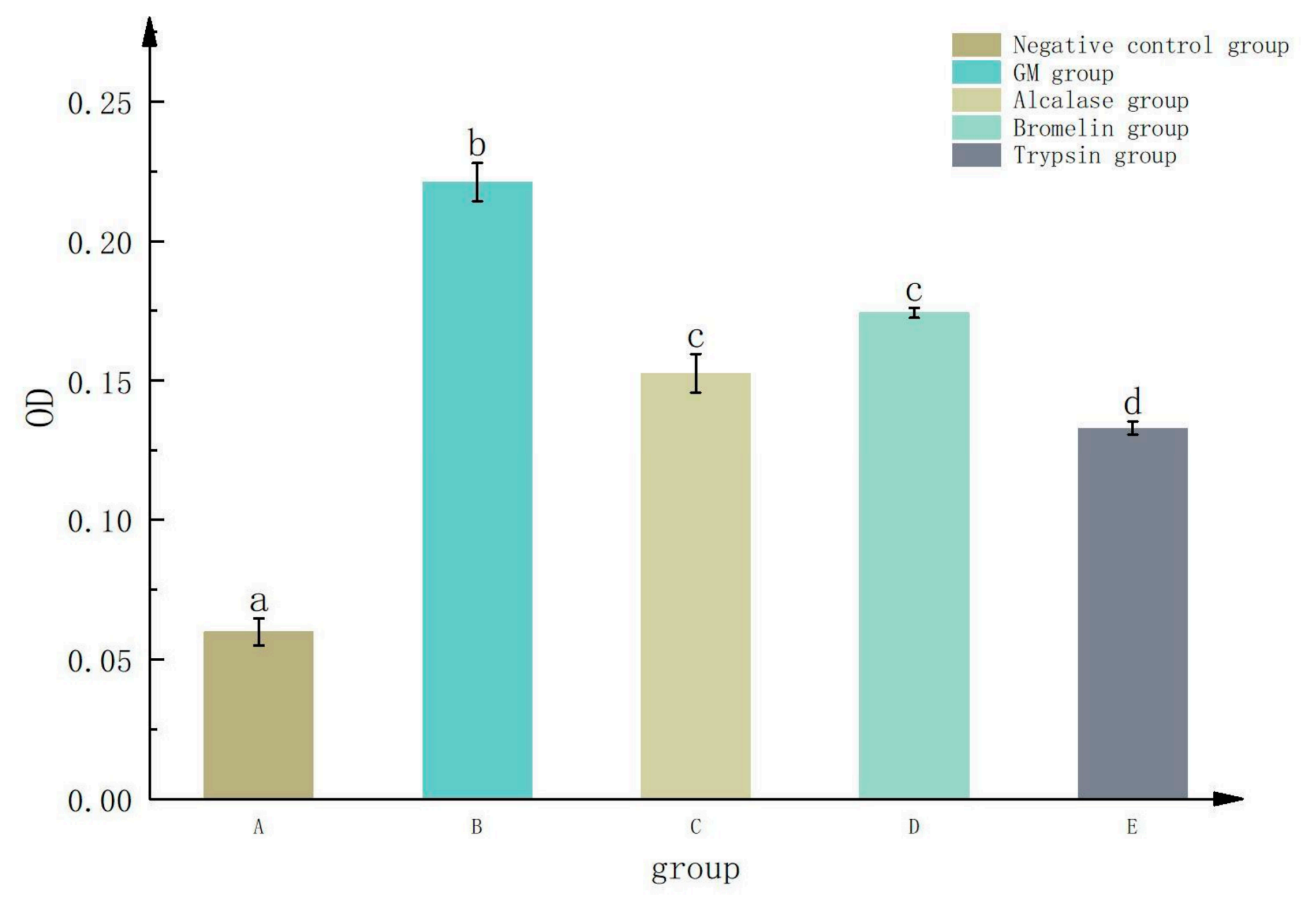
Specific IgG levels in the serum of each group of mice; different letters on the columns indicate significant differences (*p* < 0.05). Sample size *n* = 10.

As shown in Figure 3, Duncan’s multiple range test was used for statistical analysis, with different letters indicating significant differences among groups. Mice in the negative control (NC) group exhibited the lowest levels of specific IgG antibodies, which were significantly lower than those observed in all treatment groups, indicating that these mice did not develop a detectable specific humoral immune response. The goat milk total protein (GM) group exhibited the highest specific IgG antibody levels, which were significantly higher than those in the other groups, indicating successful establishment of the sensitization model and a strong specific IgG antibody response to goat milk proteins. The specific IgG antibody levels in the alkaline protease and bromelain hydrolysate groups were comparable, with no significant difference between the two groups; however, both were significantly lower than those in the GM group, indicating that hydrolysis with these two proteases effectively reduced the specific IgG antibody response induced by goat milk proteins. The trypsin hydrolysate group exhibited the lowest specific IgG antibody level, which was significantly lower than those of all other hydrolysate groups, indicating that the trypsin hydrolysate was the most effective in reducing the specific IgG antibody response.

### 2.4. Results of Total Immunoglobulin E (IgE) Measurement

**Figure 4:**
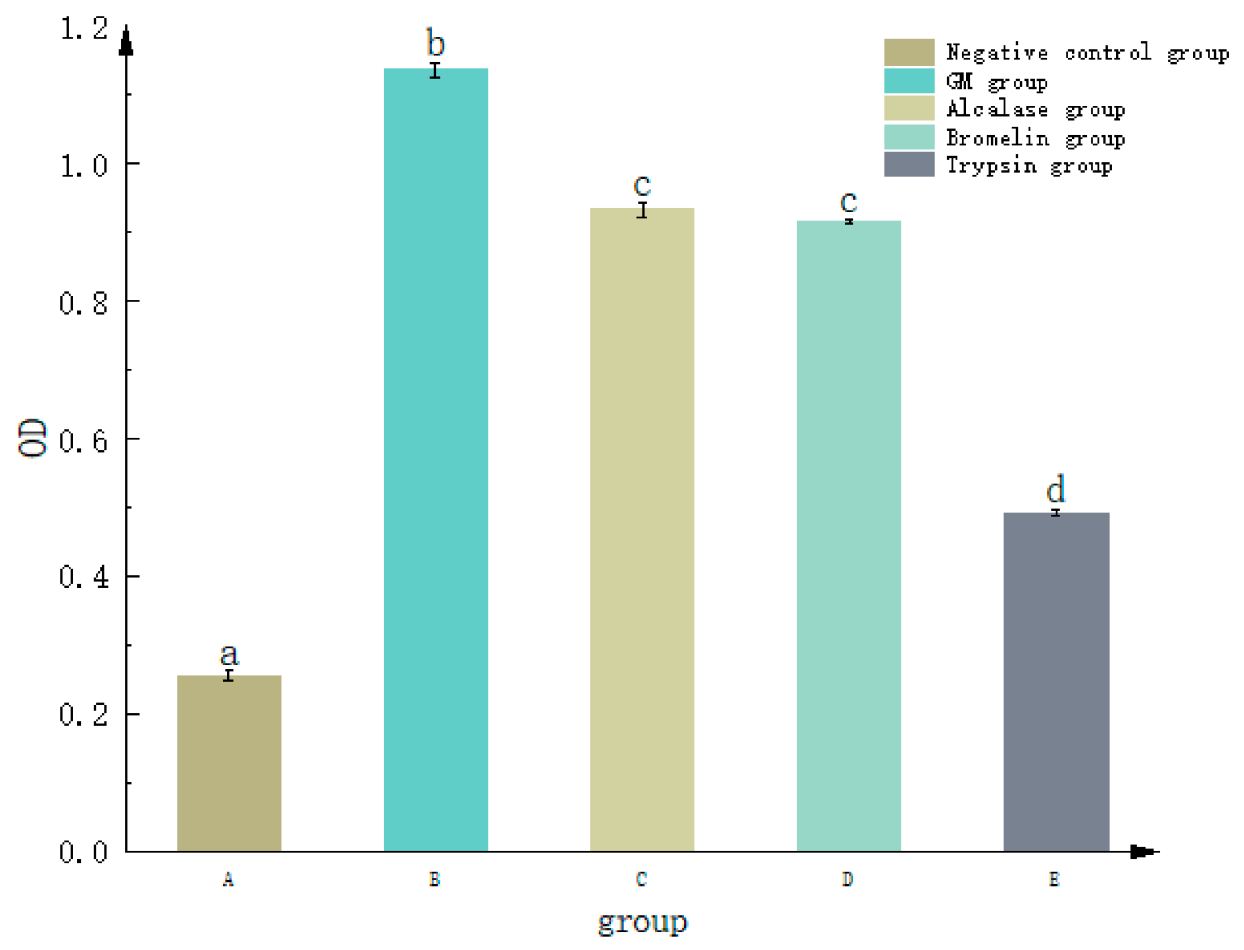
Day 21, total IgE levels in the serum of each group of mice; different letters on the columns indicate significant differences (*p* < 0.05). Sample size *n* = 10.

As shown in Figure 4, Duncan’s multiple range test was used for statistical analysis, with different letters indicating significant differences among groups. Mice in the negative control (NC) group exhibited the lowest total IgE levels, which were significantly lower than those observed in all treatment groups, suggesting that no detectable IgE-mediated immune response was elicited in this group. The goat milk total protein (GM) group exhibited the highest total IgE levels, which were significantly higher than those in the other groups, indicating successful establishment of the sensitization model and a strong IgE antibody response to goat milk proteins, a characteristic feature of Th2 cell-mediated allergic sensitization. No significant difference in total IgE levels was observed between the alkaline protease and bromelain hydrolysate groups; however, both exhibited significantly lower levels than the GM group, suggesting that both enzymatic treatments effectively attenuated goat milk protein-induced IgE production. The trypsin hydrolysate group exhibited significantly lower total IgE levels than the other hydrolysate groups, suggesting that the trypsin hydrolysate was the most effective in reducing the IgE antibody response.

### 2.5. Determination of Histamine Release

**Figure 5:**
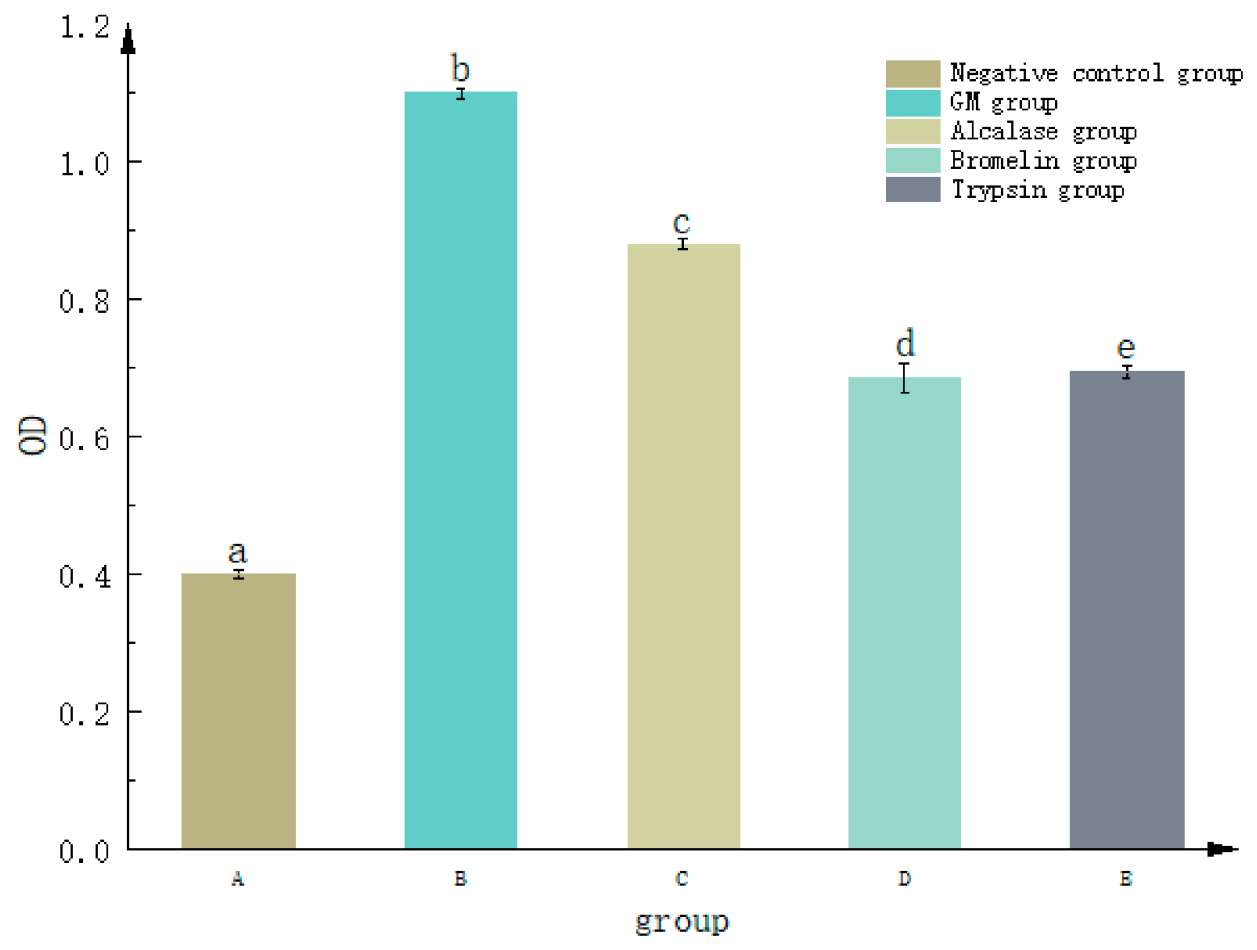
Histamine levels in serum of mice in each group; different letters on the columns indicate significant differences (*p* < 0.05). Sample size *n* = 10.

As shown in Figure 5, group comparisons were performed using Duncan’s multiple range test, with different superscript letters indicating statistically significant differences among groups. Mice in the goat milk total protein (GM) group exhibited the highest serum histamine levels, which were significantly higher than those in the NC group and all other treatment groups. These results indicate that total goat milk protein effectively elicited an allergic response, supporting the successful establishment of the sensitization model. Serum histamine levels in the alkaline protease and trypsin hydrolysate groups were significantly lower than those in the GM group, indicating that these hydrolysates elicited less histamine release than total goat milk protein. The bromelain hydrolysate group exhibited the lowest serum histamine level, which was significantly lower than those observed in all other groups, suggesting that bromelain hydrolysis was the most effective in attenuating histamine release in this cow’s milk allergy model.

### 2.6. H&Estaining Results of Jejunum in Mice

**Figure 6:**
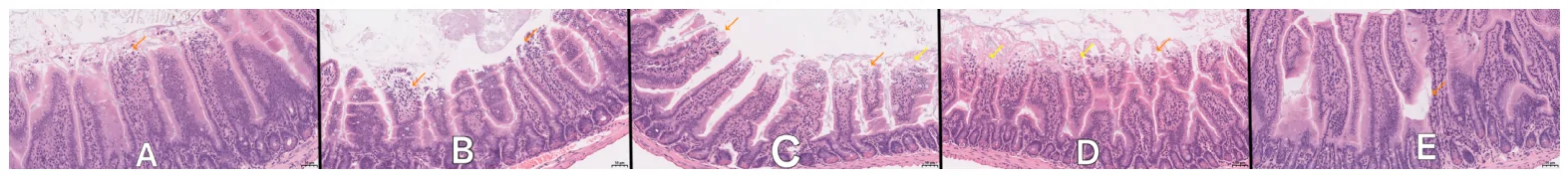
The jejunum of mice in each group was sectioned and stained with H&E. (**A**) Negative control group; (**B**) GM group (total goat milk protein group); (**C**) alkaline protease hydrolysate group; (**D**) bromelain hydrolysate group; (**E**) trypsin hydrolysate group. Orange arrows indicate epithelial cell loss at the villus tips; yellow arrows indicate karyolysis. Original magnification: 20.0×, Scale bars: 50 μm (20.0×).

H&E-stained jejunum sections from mice in the negative control (NC), goat milk total protein (GM), trypsin hydrolysate, bromelain hydrolysate, and alkaline protease hydrolysate groups were examined for histopathological changes (Figure 6). The basic histological architecture of the jejunum was generally preserved across all groups, with an adequate number of intestinal villi that were regularly arranged. The mucosal epithelium was primarily composed of columnar and goblet cells, while the intestinal glands in the lamina propria were dense and short tubular structures. The muscularis mucosae separated the glands from the submucosa, which consisted of loose connective tissue. No obvious mast cell infiltration was observed in any group.

The main differences among the groups were observed in the extent and type of epithelial injury. The NC group showed only a small amount of epithelial loss at the tips of the villi (orange arrow). The GM group exhibited more extensive epithelial loss at the villus tips, accompanied by a small amount of karyolysis (yellow arrow). The alkaline protease hydrolysate group showed lesions similar to those observed in the GM group, with relatively frequent epithelial loss and karyolysis. The bromelain hydrolysate group also exhibited more extensive epithelial loss, with a greater degree of karyolysis than that observed in the GM group. In contrast, only a small amount of epithelial loss was observed in the trypsin hydrolysate group, with no apparent karyolysis. Overall, the trypsin hydrolysate group exhibited the mildest degree of epithelial damage among all groups.

### 2.7. Results of Pathological Examination of Mouse Spleen

**Figure 7:**
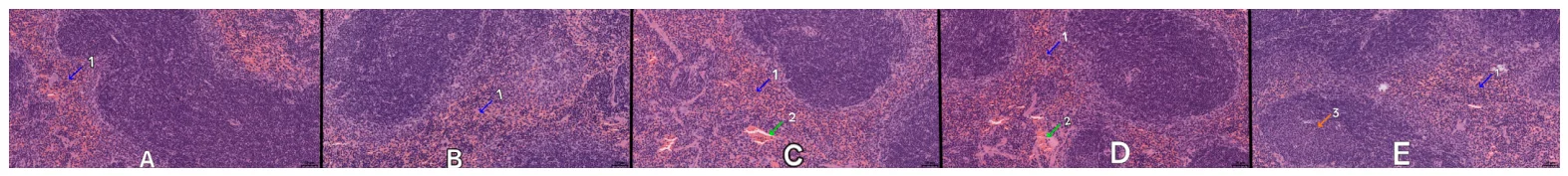
H&E staining of splenic sections from mice in each group. (**A**) Negative control group; (**B**) GM group (total goat milk protein group); (**C**) alkaline protease hydrolysate group; (**D**) bromelain hydrolysate group; (**E**) trypsin hydrolysate group. Blue arrows (1) indicate brownish-yellow pigment deposition; green arrows (2) indicate splenic sinusoidal congestion; orange arrows (3) indicate necrotic cell debris. Original magnification: 20.0×. Scale bars: 50 μm (20.0×).

As shown in Figure 7, H&E staining revealed that the basic structure of the spleen was preserved across all groups, with clearly distinguishable red pulp, white pulp, and marginal zones. The white pulp was abundant and evenly distributed, while the splenic cords and splenic sinusoids were clearly visible within the red pulp. Histopathological changes in the spleen were descriptively graded as absent (−), mild (+), moderate (++), or severe (+++), based on the extent of splenic sinusoidal congestion, sinusoidal dilation, brownish-yellow pigment deposition, and necrotic cell debris in the white pulp (Table 2).

A small amount of brownish-yellow pigment deposition was observed in the red pulp of the negative control (NC) group, with no obvious congestion, dilation, or necrosis (grade −). The goat milk total protein (GM) group exhibited mild splenic sinusoidal congestion (grade +), while the basic splenic architecture remained intact. The alkaline protease and bromelain hydrolysate groups showed mild and comparable splenic alterations (grade +), with less pronounced congestion than that observed in the GM group. In the trypsin hydrolysate group, sinusoidal congestion was less pronounced than in the GM group; however, a small amount of necrotic cell debris was observed in the white pulp of some samples (grade +). Overall, the basic splenic architecture remained intact in all groups. Although sinusoidal congestion was reduced in the trypsin hydrolysate group, the presence of white pulp necrotic cell debris indicated that mild pathological changes persisted.

## 3. Discussion

In this study, a BALB/c mouse model of cow milk allergy was established to compare the allergenic responses elicited by goat milk protein and its hydrolysates prepared with alkaline protease, bromelain, and trypsin. We assessed clinical symptoms, serum total IgE and specific IgG levels, histamine release, and histopathological changes in the jejunum and spleen.

All three enzymatic hydrolysate groups exhibited milder allergic reactions compared to the GM group, which was consistent with previous animal studies reporting differences in the sensitization levels induced by different milk proteins [26]. The clinical symptom score in the negative control group was 1.00 (0.00, 1.00), corresponding to mild scratching observed in a few mice. This may have resulted from nonspecific irritation caused by repeated gavage administration and the use of cholera toxin as an adjuvant rather than from a genuine allergic response. Regarding clinical symptom scores, all three hydrolysate groups exhibited generally milder allergic symptoms than the GM group. Notably, the bromelain hydrolysate group exhibited the lowest symptom score of 1.00 (1.00, 1.00), which was significantly lower than that of the GM group (*p* < 0.05), indicating that the bromelain hydrolysate elicited a milder allergic response in sensitized mice. Although the symptom scores of the alkaline protease and trypsin hydrolysate groups were lower than those of the GM group, these differences were not statistically significant (*p* > 0.05). These findings suggest that not all enzymatic hydrolysates elicited significantly milder allergic responses in vivo, which may be attributable to differences in their actual efficacy or the relatively small sample size. In previous in vitro experiments, the bromelain hydrolysate showed a moderate reduction in immunoreactivity against some allergens, indicating that in vitro immunoreactivity does not necessarily correlate linearly with the severity of allergic responses elicited in vivo. This finding is consistent with observations reported by Bøgh et al. [27]. This discrepancy may be attributed to the fact that the occurrence and severity of allergic symptoms depend not only on the IgE-binding capacity of allergens but also on multiple factors, including the activation threshold of mast cells, the efficiency of histamine release, and the sensitivity of target organs [28]. The results of the specific IgG and IgE antibody assays showed that antibody levels in all three enzymatic hydrolysate groups were significantly lower than those in the goat milk total protein group (GM), suggesting that enzymatic hydrolysis effectively attenuated the specific humoral immune response to goat milk proteins [29]. Although bromelain hydrolysis may have a relatively limited effect on key allergenic epitopes in vitro, its performance in vivo was superior, suggesting that its mechanism of action may be more complex. It is plausible that bromelain, a cysteine protease with broad cleavage specificity, generates a distinct peptide profile that more effectively interferes with the interaction between key allergenic epitopes and IgE or, alternatively, produces peptides with intrinsic immunomodulatory properties that suppress mast cell degranulation and effector-phase responses. Trypsin, which cleaves preferentially at lysine and arginine residues, was the most effective in reducing serum IgE and IgG levels, possibly by directly disrupting B-cell epitopes. In contrast, bromelain, with its broader cleavage specificity, may generate smaller peptides that are less capable of cross-linking IgE on mast cells while retaining the ability to modulate mast cell signaling. Therefore, the differential in vivo outcomes among the hydrolysates may not simply reflect the extent of IgE epitope destruction but may instead result from the generation of distinct peptide repertoires with varying capacities to regulate the effector phase of the allergic response. Further integration and analysis of these findings with the corresponding peptide composition and molecular weight distribution data are required. The reduction in serum histamine levels may be associated with the decreased allergenic potential of the hydrolysates. Histamine is an important inflammatory mediator released following mast cell activation, and changes in serum histamine levels can reflect the extent of mast cell activation to some extent [30]. Therefore, the lower histamine levels observed in the enzymatic hydrolysate groups may indicate attenuated mast cell activation; however, direct evidence, such as measurements of mast cell degranulation or tryptase levels, is lacking, and this possibility requires further investigation.

Histopathological analysis of the jejunum and spleen revealed differences among the groups. In the jejunum, the goat milk total protein group (GM) showed pronounced epithelial cell loss at the villus tips, accompanied by a small amount of karyolysis, indicating that the allergic response induced intestinal mucosal injury. Among the three hydrolysate groups, the trypsin hydrolysate group exhibited the mildest epithelial damage, with only limited epithelial cell detachment at the villus tips and no apparent karyolysis or nuclear disappearance. In contrast, the alkaline protease and bromelain hydrolysate groups still showed considerable epithelial injury; notably, the bromelain group exhibited more pronounced karyolysis than the GM group. In the spleen, mild splenic sinusoidal congestion was observed in the GM, alkaline protease hydrolysate, and bromelain hydrolysate groups. The trypsin hydrolysate group showed less pronounced sinusoidal congestion but exhibited a small amount of cellular necrotic debris in the white pulp. These differences in histopathological changes may be associated with differences in the molecular characteristics of the enzymatic hydrolysates, such as peptide composition, molecular weight distribution, and bioavailability. However, the absorption and tissue distribution of the different hydrolysates, as well as their direct effects on splenic immune cells, were not investigated in this study. Therefore, the mechanisms underlying these observed differences remain unclear and warrant further investigation. The semi-quantitative histopathological evaluation was performed on four randomly selected mice per group rather than the entire cohort (*n* = 10). Although this approach was sufficient to reveal the observed differences, larger sample sizes are needed in future studies to further validate the histopathological findings.

The results of the in vitro ELISA assays of the three enzymatic hydrolysates [23] were generally consistent with the findings of the present animal study, although the two evaluation approaches were not entirely concordant. In the in vitro assays, the alkaline protease hydrolysate exhibited the greatest reduction in immunoreactivity against the five major allergens. However, this superior in vitro performance was not fully reflected in vivo. The alkaline protease hydrolysate group exhibited higher serum IgE and IgG levels than the trypsin hydrolysate group, and higher clinical symptom scores than the bromelain hydrolysate group. These findings suggest that in vitro immunoreactivity does not necessarily predict the magnitude of the in vivo allergic response, highlighting the importance of animal models in comprehensively evaluating the allergic responses elicited by protein hydrolysates. This discrepancy may be explained by the fact that in vitro ELISA primarily reflects the binding capacity between antigens and antibodies and cannot fully recapitulate the complex processes of digestion, absorption, and metabolism that occur in vivo. The molecular weight distribution and peptide composition of different enzymatic hydrolysates may affect their gastrointestinal stability, intestinal permeability, and interactions with immune cells [31]. In contrast, the clinical symptoms observed in animal experiments result from the integrated effects of multiple immune cells and inflammatory mediators and therefore cannot be attributed solely to antigen–antibody binding. Therefore, combining in vitro and in vivo approaches may provide a more comprehensive assessment of the allergic responses elicited by enzymatic hydrolysates in previously sensitized animals.

In summary, all three enzymatic hydrolysates elicited weaker allergic responses in already sensitized mice to varying degrees. The trypsin hydrolysate group showed the lowest levels of specific IgG, while the bromelain hydrolysate group exhibited the lowest clinical symptom scores and serum histamine levels. These differences may be attributed to variations in protease substrate specificity and the resulting peptide composition. Therefore, the elicitation response to milk protein hydrolysates should not be evaluated based on a single indicator; instead, a multidimensional assessment integrating clinical symptoms, specific antibody responses, inflammatory mediators, and histopathological changes is necessary for a comprehensive evaluation. Because the hydrolysates were administered only during the challenge phase, this study evaluated their elicitation responses rather than their sensitizing capacity. Future studies should include hydrolysate-sensitized groups to directly assess the sensitizing potential of the hydrolysates.

## 4. Materials and Methods

### 4.1. Hydrolyzed Product Preparation

Goat milk protein hydrolysates were prepared as described in our previous study [23], and the same batch of hydrolysates was used in the present animal experiments. Briefly, goat milk total protein was hydrolyzed at a substrate concentration of 3% (*w*/*v*) with an enzyme-to-substrate ratio of 1000 U/g protein at the supplier-recommended pH and temperature for 2 h. The reaction was terminated by heating at 85 °C for 10 min. The degree of hydrolysis (DH), determined by the OPA method, was 7.07% for alkaline protease, 5.13% for bromelain, and 6.11% for trypsin.

### 4.2. Animal Experiment Scheme

The animal experiment was conducted according to the protocol described in reference [32]. Female BALB/c mice (aged 4–5 weeks) were used to evaluate the elicitation responses to goat milk protein hydrolysates in a murine model of cow’s milk allergy. Animals were purchased from vendors with laboratory animal production qualifications. All animal experiments were carried out in accordance with the institutional ethical guidelines and relevant management standards for experimental animals. All mice were acclimatized in an SPF animal room for 3 days under controlled conditions: temperature at 23 ± 3 °C, relative humidity at 40–70%, and a 12-h light/dark cycle. During this period, mice had free access to allergen-free feed and water. After acclimation, the 50 mice were randomly assigned to 5 groups (*n* = 10 per group). Each group was administered the treatment on days 0, 7, 14, 21, 28, and 35. Mice in the negative control group were intragastrically administered normal saline supplemented with 5 μg cholera toxin (AbMole Bioscience, Houston, TX, USA) as an adjuvant. The goat milk total protein group (GM) and the three hydrolysate-treated groups received 10 mg of whole milk protein solution along with 5 μg cholera toxin. On day 42, all mice were fasted overnight before being challenged. The negative control group received normal saline by intragastric gavage, whereas the goat milk total protein group (GM) was challenged with 100 mg of total goat milk protein (10-fold the dose used in the sensitization phase). The hydrolysate groups 1, 2, and 3 were intragastrically administered 100 mg of goat milk alkaline protease hydrolysate, goat milk bromelain hydrolysate, and goat milk trypsin hydrolysate, respectively.

To evaluate the extent of sensitization, retro-orbital blood was collected from each group on day 21, and serum total IgE levels were determined by ELISA to verify whether the sensitization model was successfully established.

Mice were monitored weekly for general health indicators, including physical appearance, food intake, and behavioral status. Body weights were also measured prior to each gavage administration. Allergic symptoms were monitored for 30 min following oral challenge and were scored using a 0–5 scale as follows: 0, asymptomatic; 1, scratching and rubbing around the nose and head; 2, periorbital/perioral edema, piloerection, decreased activity, or tachypnea; 3, wheezing, dyspnea, and cyanosis around the mouth and tail; 4, immobility, tremor, or convulsions; and 5, death.

On the final day (day 42), blood samples were obtained by cardiac puncture 30 min following high-dose challenge and were immediately mixed gently in centrifuge tubes without anticoagulant. Blood samples were allowed to clot for 1 h at room temperature and then centrifuged at 5000 rpm for 10 min at 4 °C, Refrigerated high-speed centrifuge (Sigma-Aldrich, St. Louis, MO, USA). The resulting serum was aliquoted and stored at −20 °C until analysis for IgE and histamine. After euthanasia, the spleen and jejunum (at 8–10 cm distal to the stomach) were excised and processed for histopathological evaluation. The degree of inflammatory cell infiltration was observed by H&E staining under a 200-fold field of view, and histopathological analysis was performed to evaluate the effector-phase allergic response after challenge.

#### 4.2.1. Determination of Specific Antibody IgG

The indirect ELISA was performed according to a modified protocol based on reference [33]. Briefly, goat milk protein was diluted to 10 μg/mL in carbonate buffer, and 100 μL of the solution was added to each well of a 96-well microplate. The plates were sealed and incubated overnight at 4 °C.

On the following day, the wells were emptied and washed three times with 200 μL of PBST washing buffer per well, for 3 min each with shaking. After washing, the plates were tapped dry to remove residual liquid. The wells were then blocked with 100 μL of blocking solution, and the plate was sealed and incubated for 1 h at 37 °C.

Test sera were serially diluted twofold from an initial dilution of 1:50 to 1:3200. Aliquots of 100 μL of the diluted sera were then added to the microplate wells and incubated for 2 h at 37 °C. The wells were subsequently washed three times with PBST and blotted dry. The plate was then incubated with HRP-labeled goat anti-mouse IgG secondary antibody (diluted 1:1000; Beijing Solarbio Science & Technology Co., Ltd., Beijing, China) at 37 °C for 1 h. Following five washes with PBST, the plate was blotted dry.

The color reaction was initiated by adding 100 μL of TMB substrate to each well, followed by incubation for 20 min in the dark at room temperature. After blue color development, the enzymatic reaction was terminated by the addition of 50 μL of 2 M H_2_SO_4_ per well, yielding a yellow color. The absorbance of each well was measured at a wavelength of 450 nm using a multifunctional microplate reader (Shanghai Kehua Experimental System Co., Ltd., Shanghai, China).

#### 4.2.2. Determination of Total Antibody IgE

Total IgE was measured using an ELISA kit (Beijing Solarbio Science & Technology Co., Ltd., Beijing, China), and the specific experimental procedures were performed according to the manufacturer’s instructions. Briefly, all reagents and samples were equilibrated to room temperature. A total of 100 µL of standards (2-fold serial dilutions) and samples were added to the wells and incubated at room temperature (25 ± 2 °C) with shaking for 120 min. After washing 4 times with 300 µL wash buffer, 100 µL of biotinylated detection antibody was added and incubated for 60 min at room temperature with shaking, followed by washing 4 times. Then, 100 µL of enzyme conjugate was added and incubated for 30 min at room temperature with shaking, followed by washing 5 times. Subsequently, 100 µL of TMB substrate was added for color development in the dark for 10–30 min, and the reaction was stopped with 50 µL of stop solution. The optical density (OD) was measured within 5 min using a microplate reader at dual wavelengths of 450 nm and 630 nm.

#### 4.2.3. Determination of Histamine in Serum

Histamine levels were measured using a commercial ELISA kit (Beijing Solarbio Science & Technology Co., Ltd., Beijing, China) following the manufacturer’s protocol.

#### 4.2.4. H&E Staining of Jejunum and Spleen in Mice

Tissue samples of the jejunum and spleen were washed with normal saline to remove residual contents and blood, and then immediately immersed in 4% paraformaldehyde at 4 °C for a minimum of 48 h to ensure optimal preservation of cellular morphology.

Following fixation, the tissue samples were processed in accordance with standard laboratory protocols for histopathological examination. The fixed tissues were trimmed to eliminate excess fat and connective tissue and then processed into uniform blocks for subsequent embedding. Tissues were dehydrated through a graded ethanol series (70%, 80%, 90%, 95%, and anhydrous ethanol; Tianjin Jindong Tianzheng Fine Chemical Reagent Factory, Tianjin, China) to ensure complete removal of water. After dehydration, tissues were cleared with xylene (Damao Chemical, Tianjin, China) and subsequently embedded in molten paraffin wax (Jingming Biotechnology, Beijing, China) to obtain standard paraffin-embedded tissue blocks. The paraffin-embedded tissue blocks were serially sectioned at 4 μm using a microtome, and the resulting sections were mounted on poly-L-lysine-coated glass slides. The slides were air-dried and then stained with hematoxylin and eosin (H&E). Hematoxylin and eosin were obtained from Zhuhai Besso Biotechnology Co., Ltd. (Zhuhai, China) and Beijing Solarbio Technology Co., Ltd., respectively. After dehydration and clearing, the sections were mounted with neutral balsam (Solarbio, Beijing, China) and allowed to dry under a fume hood.

Following section preparation, the slides were scanned with an automated digital pathology imaging system to generate high-resolution digital image files. Histopathological changes were evaluated using digital image viewing software at a magnification of 20.0×. The assessment focused on jejunal villus integrity, epithelial cell desquamation, and inflammatory cell infiltration, together with splenic white and red pulp architecture and lymphoid follicular hyperplasia. Histopathological findings were systematically described and compared among groups. Representative images of lesion areas were included as color photomicrographs to highlight the morphological differences observed across treatment groups. Four mice were randomly selected per experimental group for histopathological evaluation.

Splenic histopathological grading criteria. Splenic sections were evaluated at 20.0× magnification. Four parameters—splenic sinusoidal congestion, sinusoidal dilatation, brownish-yellow pigment deposition, and white pulp necrotic cell debris—were graded descriptively as absent (−), mild (+), moderate (++), or severe (+++) according to the proportion of affected fields and the extent of red pulp or white pulp involvement. Representative images of lesion areas were included as color photomicrographs.

### 4.3. Statistical Analysis

All experimental data were derived from the averages of three or more trials, and results are presented as means ± standard deviation. Significance analysis was performed using one-way ANOVA with Duncan’s multiple range test in SPSS 26.0 software (version 9.8.0.200), and graphs were generated using Origin 2021 (version R26.0.0.0). Mouse clinical symptom scores were analyzed using the Kruskal–Wallis H non-parametric test.

## 5. Conclusions

In this study, a BALB/c mouse model of cow milk allergy was established to evaluate the elicitation responses to goat milk protein and its hydrolysates prepared with alkaline protease, bromelain, and trypsin in already sensitized mice. All three hydrolysates elicited milder allergic responses than the total goat milk protein, but their effects differed by indicator: The trypsin hydrolysate group showed the lowest levels of specific IgG, while the bromelain hydrolysate group exhibited the lowest clinical symptom scores and serum histamine levels. These differences may be attributed to variations in protease substrate specificity and the resulting peptide composition. Overall, this study provides preliminary in vivo evidence that enzymatic hydrolysis may reduce the elicitation response to goat milk proteins in sensitized mice. Because the hydrolysates were administered only during the challenge phase, their intrinsic sensitizing capacity was not assessed. These findings support further investigation into the potential use of these hydrolysates in hypoallergenic goat milk-based formula foods, but additional studies, including hydrolysate-sensitized groups, are needed to evaluate their sensitizing potential and long-term safety.

## Figures and Tables

**Table 1:** Statistical analysis results of allergic symptom scores in mice from different treatment groups.

Group	Sample (*n*)	Median (IQR)	Mean Rank
Negative control group	10	1.00 (0.00, 1.00)	15.20
Goat milk total protein group (GM)	10	2.00 (1.00, 2.25)	36.70
Alkaline protease hydrolysate group	10	1.00 (1.00, 1.25)	25.25
Bromelain hydrolysate group	10	1.00 (1.00, 1.00)	21.05
Trypsin hydrolysate group	10	1.00 (1.00, 2.00)	29.30
Total	50		
H = 17.740	df = 4	sig. = 0.001387	

**Table 2:** Pathological Scoring: Four mice were randomly selected per experimental group for histopathological evaluation.

Group	Sample Number	Splenomegaly with Congestion in the Splenic Sinusoids	Splenic Blood Sinus Dilation	Brownish-Yellow Pigment Deposition	Necrotic Cell Debris in the White Pulp
Negative control group	G1-3	−	−	+	−
	G1-4	+	−	−	−
	G1-5	+	−	+	−
	G1-6	−	−	−	−
GM group (total goat milk protein group)	G2-13	+	−	−	−
	G2-14	+	−	+	−
	G2-15	−	−	+	−
	G2-16	+	−	+	−
Alkaline protease hydrolysate group	G3-27	+	−	+	−
	G3-28	−	−	+	−
	G3-29	+	−	+	−
	G3-30	+	−	+	−
Bromelain hydrolysate group	G4-31	−	−	−	−
	G4-32	−	−	+	−
	G4-33	−	−	+	−
	G4-34	+	−	+	−
Trypsin hydrolysate group	G5-42	+	−	+	+
	G5-43	−	−	+	−
	G5-44	−	−	+	+
	G5-45	−	−	+	−

## Data Availability

The original contributions presented in this study are included in the article. Further inquiries can be directed to the corresponding author.

## Abbreviations

BSA	Bovine serum albumin
PB	Phosphate buffer
NC	Negative control
CT	Cholera toxin
OD	Optical density
HE	Hematoxylin-eosin
